# Multisite Fe^3+^ Luminescent Centers in the LiGaO_2_:Fe Nanocrystalline Phosphor

**DOI:** 10.3390/molecules30112331

**Published:** 2025-05-27

**Authors:** Ajeesh Kumar Somakumar, Ivo Romet, Agnieszka Grabias, Marcin Kruk, Shusaku Hayama, Damian Wlodarczyk, Justyna Barzowska, Yadhu Krishnan Edathumkandy, Eduard Feldbach, Puxian Xiong, Yaroslav Zhydachevskyy, Monika Trzaskowska, Hanka Przybylinska, Andrzej Suchocki

**Affiliations:** 1 Institute of Physics, Polish Academy of Sciences, Aleja Lotników 32/46, 02-668 Warsaw, Poland; wlodar@ifpan.edu.pl (D.W.); yadhu@ifpan.edu.pl (Y.K.E.); zhydach@ifpan.edu.pl (Y.Z.); przyby@ifpan.edu.pl (H.P.); 2 Institute of Physics, University of Tartu, W. Ostwald Str. 1, 50411 Tartu, Estonia; ivo.romet@ut.ee (I.R.); eduard.feldbach@ut.ee (E.F.); 3 Łukasiewicz Research Network—Institute of Microelectronics and Photonics, Aleja Lotników 32/46, 02-668 Warsaw, Poland; agnieszka.grabias@imif.lukasiewicz.gov.pl; 4 Institute of Human Nutrition Sciences, Warsaw University of Life Sciences, Nowoursynowska 159c, 02-776 Warsaw, Poland; marcin_kruk@sggw.edu.pl (M.K.); monika_trzaskowska@sggw.edu.pl (M.T.); 5 Diamond Light Source, Harwell Science & Innovation Campus, Didcot OX11 DE, UK; shusaku.hayama@diamond.ac.uk; 6 Institute of Experimental Physics, Faculty of Mathematics, Physics and Informatics, University of Gdansk, Wita Stwosza 57, 80-952 Gdańsk, Poland; justyna.barzowska@ug.edu.pl; 7 Department of Electrical and Electronic Engineering, The University of Hong Kong, Hong Kong 999077, China; pxxiong@hku.hk

**Keywords:** gallates, multisites, iron doping, luminescence

## Abstract

An extensive experimental study of trivalent iron (Fe^3+^) ions in orthorhombic lithium gallate nanocrystals was undertaken. Various spectroscopic methods, such as Raman spectroscopy, extended X-ray absorption fine structure, the Mössbauer effect, electron paramagnetic resonance, photoluminescence, thermoluminescence, and cathodoluminescence were used to investigate the synthesized phosphor. This study revealed the existence of multiple Fe^3+^ sites, out of which the tetrahedral sites are preferentially occupied. Extensive optical studies showed that the Fe^3+^ doped lithium gallate phosphor is a promising candidate for various luminescence and thermoluminescence-related applications in the near-infrared regime.

## 1. Introduction

Lithium metagallate (LiGaO_2_) and lithium pentagallium oxide (LiGa_5_O_8_) are the two important ultra-wide-bandgap ternary metal oxides formed by lithium and gallium. Both compounds exhibit different structural and optical properties. LiGaO_2_ is considered to be one of the best lattice-matched substrate materials for GaN growth and frequency conversion application [1]. It is also important as an anode material in lithium-ion batteries with very good cycling stability [2]. Recently, p-type conductivity was reported for LiGa_5_O_8_, which is thus the widest band-gap oxide semiconductor known to date with p-type conductivity [3]. If doped with suitable activator ions, both phosphors exhibit significant mechanoluminescence and thermoluminescence properties at ambient temperatures [4,5]. LiGaO_2_ crystallizes in the orthorhombic phase with space group *Pna*2_1_, which is a distorted wurtzite-like structure. LiGa_5_O_8_ mainly crystallizes a cubic primitive phase with the *P*4_3_32 space group [6].

The near-infrared (NIR) emitting Fe^3+^-doped LiGaO_2_ and LiGa_5_O_8_ are some of the best phosphor materials for diverse applications, such as a potential alternative to Cr^3+^ based phosphors in bioimaging, night vision, and optical sensors, and also showing promise as thermoluminescent materials for radiation dosimetry and environmental monitoring [7,8]. Some other studies have also indicated that these types of ceramic phosphors are well applicable in several biological fields due to their biocompatible nature [7,9]. Both oxides show broadband emission in the near-infrared range at room temperature related to the ^4^T_1_(G)→^6^A_1_(S) transitions between the two lowest crystal field levels of Fe^3+^. In a recent publication [10], we investigated the luminescent and mechanoluminescent properties of the dominant Fe^3+^ emitting center in the LiGaO_2_:Fe phosphor grown by solid-state reaction. The present work focuses on multi-site occupancy of Fe^3+^ ions in the sample studied. Special attention is devoted to identifying the nature of different Fe^3+^ centers and their influence on the optical properties of this material. For this purpose, a few advanced techniques are used, such as extended X-ray absorption fine structure (EXAFS), electron paramagnetic resonance (EPR), and Mössbauer spectroscopies. The thermo-stimulated luminescence (TSL) and cathodoluminescence (CL) properties of the phosphor are also investigated, and the results show the potential of NIR-emission-based applications of the LiGaO_2_:Fe^3+^ phosphor. We also studied the anti-bacterial properties of LiGaO_2_:Fe^3+^. The results reported in the Supporting Information (Text S1) do not exhibit such potential for this material against the selected types of bacteria.

## 2. Results and Discussion

### 2.1. Structural and Raman Studies

Previous X-ray powder diffraction studies of the synthesized material [10] have shown that the dominant phase is orthorhombic LiGaO_2_ with the *Pna*2_1_ space group (card number PDF 04-007-9560), identical to the structure refined by Marezio [11]. In addition, about 0.8% of the LiGa_5_O_8_ impurity phase (card number PDF 04-002-8232) was detected. The formation of this phase probably arose due to the relatively high calcination temperature, since the transformation from LiGaO_2_ to LiGa_5_O_8_ was shown to start already at ≈1373 K (1100 °C) [12].

**Figure 1:**
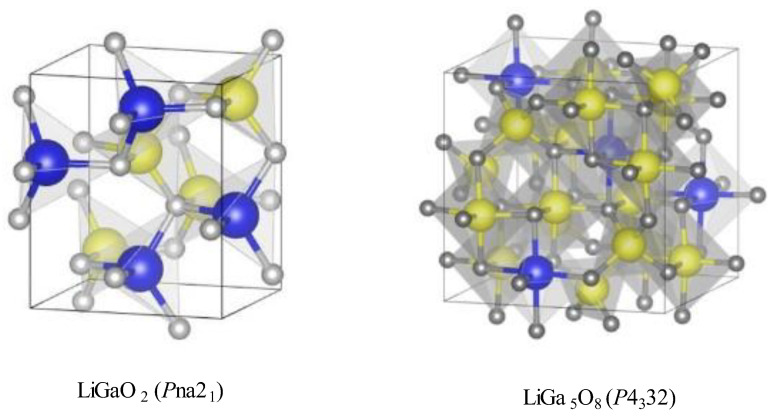
Crystal structures of LiGaO_2_ and LiGa_5_O_8_ phases (blue balls−lithium, yellow−gallium, grey−oxygen).

The LiGa_5_O_8_ impurity phase has a cubic spinel structure (space group *P*4_3_32) with a unit cell parameter of a = 8.203 Å (card number PDF 04-002-8232). The LiO_6_ octahedra share their corners with six GaO_4_ tetrahedra and their edges with the GaO_6_ octahedra. The structure is shown in Figure 1 in comparison to that of LiGaO_2_. The Fe^3+^ ion replaces Ga^3+^ in the tetrahedral sites of LiGaO_2_, while in the LiGa_5_O_8_ impurity phase, Fe^3+^ can replace Ga^3+^ both in the tetrahedral (A) and the octahedral (B) sites without much change in the structure, since both Fe^3+^ and Ga^3+^ have very close Shannon ionic radii in tetrahedral as well as octahedral coordination.

**Figure 2:**
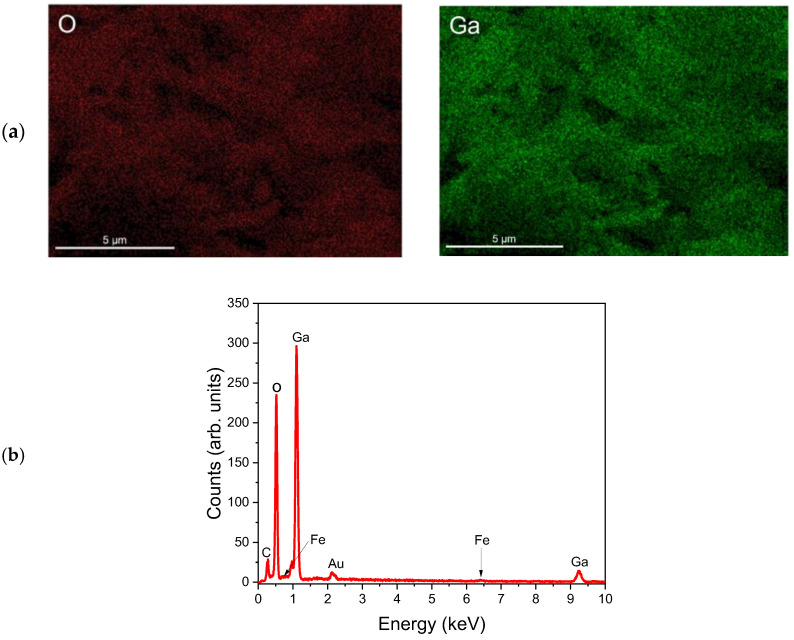
EDS maps of oxygen (K-edge) and gallium (L-edge) elements in LiGaO_2_:Fe^3+^ (**a**) and the EDS spectrum (**b**). Peaks related to C and Au come from the contamination with atmospheric CO_2_ and the electrical conducting layer of the sample, respectively.

The elemental maps in Figure 2a show a uniform distribution of Ga and O ions in the sample. Due to the very low concentration, iron cannot be visualized in the maps. However, the EDS spectrum shown in Figure 2b confirms its presence.

**Figure 3:**
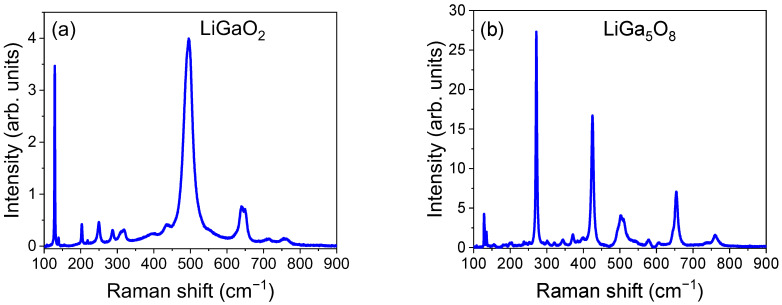
Raman spectra of the synthesized LiGaO_2_:Fe sample containing the main, orthorhombic LiGaO_2_ phase (**a**) and the cubic LiGa_5_O_8_ impurity phase (**b**).

Figure 3 presents the room temperature Raman spectra of the main LiGaO_2_:Fe^3+^ phase (a) and the LiGa_5_O_8_:Fe^3+^ impurity phase (b), respectively. The spectra were collected by focusing the laser beam on two different places of the sample. Both closely match the Raman peaks reported for undoped LiGaO_2_ [13,14] and LiGa_5_O_8_ [14] powder samples, as listed in Table 1. In addition, some weak peaks at 139, 135, 219, 319, 397, 556, and 714 cm^−1^ are detected, which were not reported previously in the Raman spectra [13,14]. They may be due to local phonons related to Fe doping or defects.

### 2.2. XAS, EXAFS, Mössbauer & EPR Measurements

**Figure 4:**
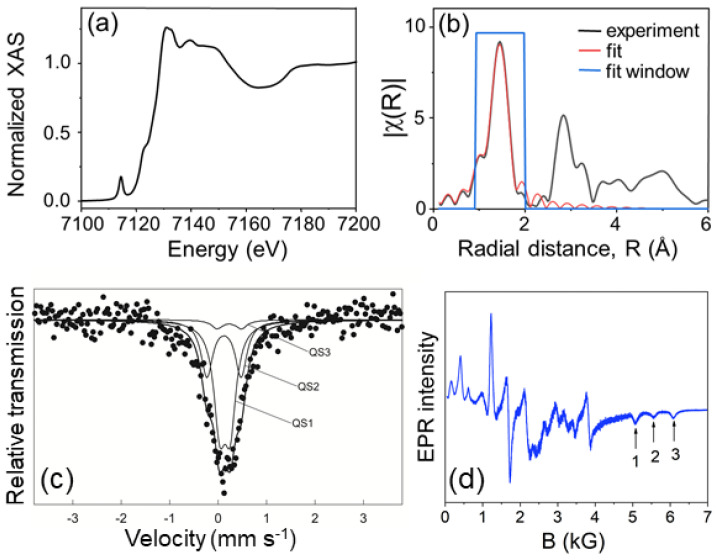
(**a**) Normalized K−edge XAS spectrum of Fe^3+^ in LiGaO_2_. (**b**) Nearest neighbor shell fit (red line) to the Fourier−transform EXAFS data (black line). The fitting range is shown by the blue window. (**c**) Mössbauer spectrum of LiGaO_2_:Fe^3+^ fitted with three quadrupole doublets (QS). (**d**) EPR spectrum at 3 K after subtraction of the background.

To obtain more information on the coordination of Fe ions in the LiGaO_2:_Fe^3+^ sample, we performed XAS and EXAFS measurements. The XAS spectrum recorded near the Fe K-edge is shown in Figure 4a. In this region, the data were taken in steps of 0.3 eV with the monochromator calibrated with an iron foil at the Fe K-edge of 7112 eV. The pre-edge feature at 7114.5 eV arises from 1s → 3d transitions. The shape of this single peak and its relatively strong intensity indicate that 4p and 3d orbitals of Fe are mixed, and the local site symmetry of Fe is unlikely to be centrosymmetric [15]. The absorption in the EXAFS region was measured with a constant wavenumber step of 0.04 Å^−1^. The spectrum was then normalized with Athena software and processed further in Artemis [16]. The obtained EXAFS spectrum is shown in Figure 4b. Only the first shell of the EXAFS data was fitted using the crystalline structure of LiGaO_2_, with Fe^3+^ replacing one of the Ga^3+^ sites. The Ga–O bond length for LiGaO_2_ has been previously reported to be 1.835 ± 0.004 Å [11]. According to this simple model, the first shell around the Fe atom consists of four oxygen atoms at 1.845 Å. This value is close to the Fe–O bond length of 1.82 ± 0.02 Å reported for LiAlO_2_ (an isomorphic material to LiGaO_2_) doped with Fe^3+^ [15]. The first shell was fitted in the range of 0.95 to 2.0 Å with the wavenumber range of 2.5 to 12.3 Å^−1^.

The EXAFS data confirm that the local environment around Fe is predominantly tetrahedral. No Fe^3+^ occupancy in octahedral sites related to the LiGa_5_O_8_ impurity phase was detected.

The sample was further analyzed using Mössbauer spectroscopy. The weak absorption (below 1%) in the Mössbauer spectrum shown in Figure 4c is due to the low natural content of the ^57^Fe isotope. The signal-to-noise ratio was improved by a sufficiently large number of counts per channel. Hence, the spectrum reveals a distinct hyperfine structure originating from several nonequivalent Fe^3+^ iron sites. The spectrum was fitted with three components. Two substantial quadrupole doublets (QS_1_ and QS_2_) reveal a similar isomer shift in the 0.23–0.24 mm/s range. The values below 0.30 mm/s can be connected with Fe^3+^ ions in tetrahedral coordination, which agree with the substitution of iron ions for Ga^3+^ in the LiGaO_2_ structure. It is known that Ga^3+^ ions occupy only one site in the LiGaO_2_ structure. The presence of two doublets with a similar isomer shift (IS) but different quadrupole splitting values (QS_1_ = 0.22 mm s^−1^, QS_2_ = 0.70 mm s^−1^) indicates that Fe^3+^ ions occupy structural positions with a higher (QS_1_) and a lower (QS_2_) local symmetry within the same phase. It is worth noting that the high symmetry Fe^3+^ ions have the most significant contribution to the total spectral area of about 61%. An additional spectral component, like the QS_2_ doublet, is often observed in the Mössbauer spectra measured for nanoparticles of different iron-doped oxides. It is associated with a relatively significant contribution of iron ions in distorted surface regions [17]. The third minor quadrupole doublet has an isomer shift of 0.34 mm s^−1^, typical for Fe^3+^ ions in octahedral coordination, e.g., in the LiGa_5_O_8_ structure, which was reported to have a quadrupole splitting comparable to the present case (QS_3_ = 0.50 mm s^−1^) [18]. The relative spectral contribution of this minor component does not exceed 5%.

However, the relatively large noise in the spectrum in Figure 4c is related to a weak absorption effect due to the low natural content of the ^57^Fe isotope in the sample. At first, the spectrum was fitted with only two QS components. However, the residuum and the distribution of quadrupole splitting values calculated for the entire spectrum revealed that the fit could be improved by introducing the QS3 doublet. Such a fitting of the spectrum gave the best value of the chi-square.

The vast majority of Fe^3+^ ions substitute for Ga^3+^ at the tetrahedral sites. A minor contribution of Fe^3+^ at the octahedral sites was observed, most probably due to the presence of the LiGa_5_O_8_ phase. Another Mössbauer study of LiAlO_2_ doped with iron (Fe^3+^) also shows a comparable IS value of about 0.16 mm s^−1^ and a QS value of 0.623 mm s^−1^ [15]. However, in the case of single crystals, Fe^3+^ occupies only one tetrahedral site.

The EPR measurements were performed to check whether Fe^3+^ ions occupy more than one lattice site in the LiGaO_2_ sample. The powder spectrum collected at 9.5 GHz and 3 K is shown in Figure 4d. Here, the practically temperature-independent broad background was subtracted (the spectrum in Figure 4d is the difference between experimental data collected at 3 and 50 K) to emphasize the narrow, iron-related lines. The spectrum shows a rich number of singularities, already at first glance, exceeding the number expected for a single site. Single site, high spin X band powder spectra have been analyzed before, based on spin Hamiltonian parameters obtained independently from the high frequency (95 GHz) analysis and/or single crystal experimental data [19,20,21]. Because of considerable zero field splitting mixing the spin wave functions for $\left.{|M}_{S}\right\rangle$, (M_S_ = −5/2, −3/2,…, 3/2, 5/2) at low magnetic fields, and for B, this additional information was necessary to attribute the observed singularities to the extremes and the crossing points in the angular dependencies of characteristic transitions. Attempts to obtain spin Hamiltonian parameters of single, high-spin centers from X-band powder spectra alone have not been reported. In our case, we cannot rely on the support of single crystal or high-frequency data (at high magnetic fields the wave functions reflect accurate spin projections for $\left.{|M}_{S}\right\rangle$), as both the single crystals and the necessary high-frequency equipment are lacking. Therefore, we only present a qualitative analysis aiming to eliminate impossible interpretations of the experimental data.

The Ga and Li sites in LiGaO_2_ and the octahedrally coordinated Ga sites in LiGa_5_O_8_ have all orthorhombic point symmetry. Therefore, the spin Hamiltonian for Fe^3+^ (including only the second-order crystal field terms) is given by the following equation:

(1) $$H=\mu_{B}S\cdot g\cdot B+S\cdot D\cdot S,$$

where the first term is the Zeeman interaction (here S = 5/2, $\mu_{B}$ is the Bohr magneton, and **g** is the spectroscopic splitting tensor), and the second term describes the second-order crystal field splitting of the energy levels. The components of the **D** tensor fulfill the relation as follows:

D_x_ + D_y_ + D_z_ = 0. (2)

The tetrahedrally coordinated Ga site in LiGa_5_O_8_ has a higher C_3_ point symmetry, for which D_x_ = D_y_.

The only singularities in the powder spectrum that can be unambiguously assigned are the three negative peaks at high magnetic fields labeled “1”, “2”, and “3” in Figure 4d. For D_z_ > 0, they correspond to −3/2↔ −5/2 transitions (for D_z_ < 0 to +3/2↔ +5/2). In the first step, we definitely excluded the possibility that all three belong to the same center, since no choice of parameter signs fulfills Equation (2). Also, ascribing any two of the peaks to one center led to the appearance of a prominent structure in the powder simulation that is not observed in the experimental spectrum. Thus, we conclude that we deal with three different centers of orthorhombic symmetry, which agrees with the findings from the Mössbauer effect investigations. Assuming g_z_ = 2, the only parameters that could be unambiguously determined are: |D_z_| = 560, 720, and 896 G for centers “1”, “2”, and “3”, respectively. We assign the most intense center “1” to isolated Fe^3+^ ions on Ga sites in the LiGaO_2_ phase and the other two to Fe^3+^ ions with some defects in their vicinity.

### 2.3. Low-Temperature Photoluminescence

**Figure 5:**
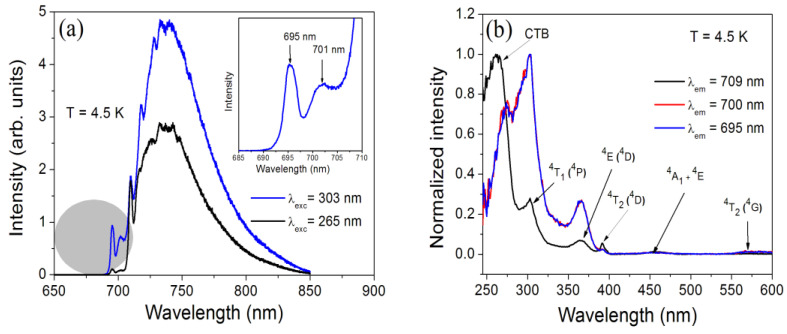
Emission (**a**) and excitation (**b**) spectra of LiGaO_2_:Fe^3+^ at 4.5 K. The excitation (λ_exc_) and emission (λ_em_) wavelengths are indicated in the figure. The inset in (**a**) shows an expanded view of the short wavelength part.

The luminescence of Fe^3+^ at 4.5 K is presented in Figure 5a for two excitation wavelengths: 303 nm (blue line) and 265 nm (black line). The dominant spectrum with the peak at 743 nm and the zero phonon line (ZPL) at 709 nm was reported previously and identified as stemming from the ^4^T_1_(G)→^6^A_1_(S) transition of Fe^3+^ ions occupying Ga sites in LiGaO_2_ [10]. Two additional features are observed at shorter wavelengths—a ZPL peak at 695 nm and a phonon replica at 701 nm. The intensity of these peaks increases relative to the 709 nm line under direct excitation to the ^4^T_1g_(^4^P) state (303 nm) as compared to indirect excitation via the charge transfer band (CTB) at 265 nm. This is also visible in the excitation spectra shown in Figure 5b. Apart from the different excitation efficiencies for the 695 and 709 nm emission lines, the positions of the Fe^3+^ excited states are practically identical, which suggests that we deal with iron ions in only slightly different surroundings. The assignment of the 695 nm center to a tetrahedrally coordinated Fe^3+^ ion in the LiGa_5_O_8_ impurity phase can be excluded, since the ZPL peak should occur at 662 nm [22].

**Figure 6:**
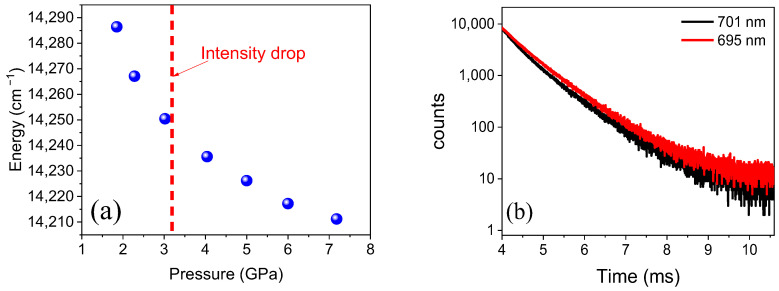
(**a**) Pressure dependence of the 695 nm emission line. (**b**) Decay kinetics recorded at 695 and 701 nm.

Confirmation that the 695 nm ZPL stems from iron ions in the main LiGaO_2_ phase is obtained by investigating its behavior under hydrostatic pressure, as shown in Figure 6a. This behavior is identical to that for the isolated Fe^3+^ center in LiGaO_2_ reported previously [10]. With increasing pressure, the luminescence peak shifts to lower energy, and its intensity decreases monotonically up to 3 GPa. Above 3 GPa the intensity drastically decreases due to the orthorhombic-to-trigonal phase transition of LiGaO_2_ [23] (see Figure S3 in the Supplementary Information). The peak disappears above 7 GPa, which we attributed to luminescence quenching due to amorphization of the LiGaO_2_ phase [10].

Thus, we assign the PL lines at 695 and 701 nm to iron ions with some defects in their vicinity. The low-temperature decay kinetics of the two peaks measured under 303 nm excitation shown in Figure 6b supports such an assignment. The decay times obtained from the fit with a double exponential function yield components with short recombination times of 0.34 ms and 0.31 ms and components with longer times of 0.76 ms and 0.73 ms for the 695 nm and 701 nm peaks, respectively. While the former recombination times are of the same order as those determined for the ZPL at 709 nm and attributed to Fe^3+^ ions at sites close to the nanocrystal surface, the latter is an order of magnitude shorter than those for isolated Fe^3+^ ions inside the nanocrystals [10]. Evidently, the presence of defects in the vicinity of iron strongly influences the recombination rate.

**Figure 7:**
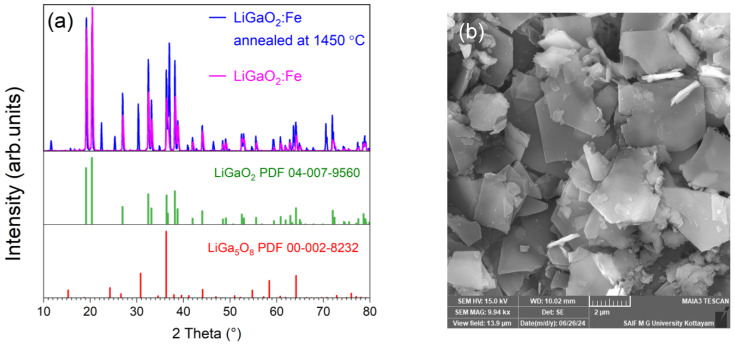
XRD patterns measured for two samples LiGaO_2_:Fe, as prepared and annealed at 1450 °C, along with reference patterns for the identified phases: LiGaO_2_ (PDF 04-007-9560) and LiGa_5_O_8_ (PDF 00-002-8232) (**a**) and SEM micrograph (**b**) of the annealed sample.

Finally, to increase the content of the LiGa_5_O_8_ impurity phase, the synthesized sample was annealed at 1450 °C for 4 h under atmospheric conditions. Previous thermal stability studies have shown that LiGaO_2_ is stable up to 1595 °C [24]. An analysis of the XRD pattern presented in Figure 7a revealed that the content of LiGa_5_O_8_ increased from 0.8% [9] to 27%. After annealing, the crystallite sizes estimated using the Debye–Scherrer equation increased from 32 nm [10] to 47.72 nm. Also, the morphology changed from the microrod-like structures in the as-grown material (see Figure S1a) to flake-like shapes of several micrometer diameters, as shown in Figure 7b. However, the shape of the photoluminescence spectrum remained the same as before annealing (see Figure S4 in the Supplementary Information). The slight difference associated with the broader part of the spectrum of the sample annealed at 1450 °C most probably comes from the different morphology of the samples. In particular, no additional emission from Fe^3+^ in the tetrahedrally coordinated Ga sites in LiGa_5_O_8_ was detected. This indicates that Fe^3+^ preferentially occupies octahedrally coordinated Ga sites in the impurity phase, which agrees with the literature [22]. Such centers only emit very weakly, and no luminescence is observed even for much higher iron concentrations [22].

### 2.4. Thermo-Stimulated Luminescence & Cathodoluminescence

**Figure 8:**
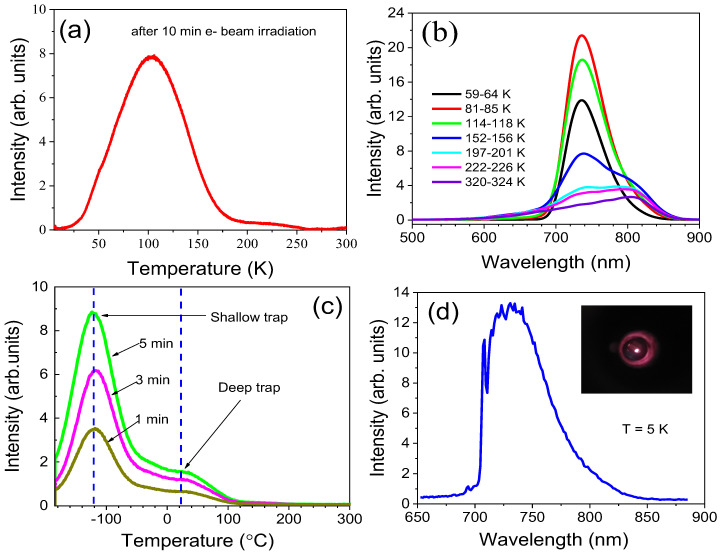
(**a**) TSL glow curve and (**b**) TSL spectra after irradiation with an electron beam. (**c**) TSL glow curves for different times of UV irradiation. (**d**) CL spectrum of LiGaO_2_:Fe^3+^ at 5 K. Inset: image of the NIR CL emission from the phosphor.

The TSL glow curve monitoring the Fe^3+^ emission shown in Figure 8a was measured with a heating rate of 0.1 K/s after 10 kV electron beam irradiation. It consists of two peaks, a strong one at 100 K and a weaker one at 220 K, which indicates the existence of at least two kinds of traps in the material. Similar traps were reported in undoped LiGaO_2_ [25], which suggests their intrinsic origin. Possible candidates are oxygen vacancies and antisite defects [5,7]. Figure 8b presents the emission spectra recorded during warming. As can be seen, in addition to Fe^3+^ emission, a luminescence band peaked at about 820 nm appears around 150 K. We interpret that this band is due to donor–acceptor recombination of intrinsic defects present in the matrix after electron irradiation. Similar TSL peaks to those shown in Figure 8a are visible after 254 nm excitation (Figure 8c). The peaks are shifted to higher temperatures because of the 10 times higher heating rate of 1 K/s. The increase of TSL intensity after prolonged irradiation (1, 3, and 5 min) indicates that both the shallow and deep traps are not fully populated.

Figure 8d shows the cathodoluminescence spectrum of LiGaO_2_:Fe^3+^ at 5 K. Like in low-temperature photoluminescence, the characteristic phonon lines related to the main Fe^3+^ center are visible in the CL spectrum. The ZPL at 695 nm is very weak compared to the ZPL at 709 nm, confirming that this Fe-defect center is less efficiently excited with the above band-gap irradiation. This is also observed under 160 nm excitation (Figure S2). We note, however, that at high enough excitation energies (Figure 8d and Figure S2), an additional ZPL at 657.6 nm appears, with a similar intensity to that of the 695 nm ZPL. It agrees with the observation of three Fe-related centers in EPR.

## 3. Experimental Section

### 3.1. Materials

The 0.25 mol% Fe doped LiGaO_2_ sample was synthesized by high-temperature solid-state reaction of LiCO_3_, Ga_2_O_3_, and Fe_2_O_3_ in stoichiometric ratios. Li_2_CO_3_ (99.99%, Aladdin, Shanghai, China), Ga_2_O_3_ (99.99%, Aladdin, Shanghai, China), and Fe_2_O_3_ (99.99%, Macklin, Shanghai, China) were used as raw materials. The target phosphors were designed in the nominal chemical composition of LiGa_1 x_O_2_:_x_Fe^3+^ (x = 0.0025) considering the preferred occupancy of Ga sites by Fe^3+^ ions. The starting materials were weighed accurately and mixed thoroughly in an agate mortar. The powdered precursor materials were first heated to 900 °C for two hours in an alumina crucible and then calcined for four hours at 1150 °C, as described in detail in our previous publications [7,10]. The final product contained 99.2% LiGaO_2_ and 0.8% LiGa_5_O_8_ phases [10]. The research reported here is related to the sample containing 0.25% Fe^3+^, which exhibited the highest luminescence efficiency among the materials with an iron concentration between 0.1 and 2 mol.% [7].

### 3.2. Experimental Methods

Photoluminescence (PL) excitation, emission, and decay studies were performed on a Horiba fluorolog-3 modular spectrofluorometer with a 450 W xenon lamp as the excitation source. For excitation in the region of vacuum UV (VUV), a 150-W deuterium discharge lamp (Hamamatsu L11798) and a McPherson 234/302 (McPherson Inc, Chelmsford, MA, USA) vacuum monochromator were used. Grating monochromators Andor SR 303i-B (Oxford Instruments, UK) equipped with H8259-02 (Hamamatsu Photonics, Tokyo, Japan) photon counting heads were used for PL detection. Thin pellets were mounted on a sample holder of an ARS closed cycle helium cryostat for measurements in the UV–Vis and vacuum UV (VUV) spectral regions, respectively. All emission spectra were corrected for the monochromator spectral efficiency and spectral sensitivity of the detectors.

The cathodoluminescence equipment included an ARS closed-cycle cryostat (Advanced Research Systems, Macungie, PA, USA) (5–400 K) and two monochromators that cover the spectral range from NIR (1700 nm) to VUV (110 nm): an in-house vacuum double monochromator with a Hamamatsu photomultiplier R6836 and an ARC SpectraPro 2300i monochromator (Princeton Instruments, Teledyne, Waterloo, ON, Canada) with a variety of gratings and detectors. The Kimball Physics EGG-3101 electron gun (Kimball Physics, Wilton, NH, USA) was utilized in both pulsed (10 ns, 5 kHz) and continuous modes.

Thermo-stimulated luminescence measurements were carried out using a liquid nitrogen-cooled FTIR 600 temperature controller from Linkam Scientific (Redhill, UK). Before measurements, the samples were either irradiated at 254 nm with a mercury lamp or electron irradiated at 10 kV.

The Raman spectra were recorded at room temperature with an S&I Gmbh MonoVista CRS+ Raman spectrometer (Anroechte, Germany) with a monochromator from Acton Princeton featuring a holographic grating of 2400 grooves/mm and a nitrogen-cooled CCD detector. The 532 nm laser line was used as the excitation source.

Field Emission Scanning Electron Microscopy (FE-SEM) and Energy Dispersive Spectroscopy (EDS) measurements were performed on a TESCAN setup (model: MAIA3 XMH) equipped with a Schottky FE gun source and ultra-high resolution imaging capabilities with a secondary electron detector (Brno, Czech Republic). The X-ray diffraction (XRD) experiment was performed with a BRUKER D2 PHASER (Preston VIC, Canada) employing Cu Kα radiation and operated at 30 kV and 10 mA. The XRD pattern was collected with a scan step of 0.02° and an acquisition time of 1 s per step. The crystal phase analysis was performed using DIFFRAC.EVA V4.1 evaluating software from BRUKER and ICDD PDF-4 database (2023). Applied semi-quantitative phase analysis is based on comparing the reflection intensities assigned to identified phases and the I/ICOR parameters from cards of PDF standards for these phases.

The X-ray absorption spectroscopy (XAS) and EXAFS measurements at the Fe K-edge were collected in fluorescence mode at the synchrotron I20-scanning Diamond Light Source, Oxfordshire, UK. The beamline is equipped with a four-bounce Si (111) monochromator [26] and a multi-element solid-state Ge detector. The beam size at the sample was 400 × 300 μm^2^. The spectra were taken at room temperature from a powder LiGaO_2_:Fe sample pressed into a pellet.

The ^57^Fe Mössbauer spectroscopy measurement was performed in transmission geometry at room temperature using a ^57^Co-in-Rh source. The isomer shift values are given relative to the α-Fe standard.

The EPR spectrum was recorded at 3 K with using a BRUKER EMX plus spectrometer operating at 9.5 GHz.

High-pressure measurements were performed in a diamond anvil cell from easyLab Technologies Ltd. (Diksmuide, Belgium) using a mixture of methanol and ethanol (5:1 ratio) as a pressure transmitting medium and ruby as a pressure gauge. The PL spectra were excited with the 275.4 nm Ar-Ion laser line and recorded with a Horiba Jobin-Yvon FHR 1000 monochromator (Glasgow, UK). More details can be found in our previous publications [10,27].

## 4. Summary and Conclusions

Extensive spectroscopic studies of the 0.25 mol% iron-doped LiGaO_2_ phosphor were conducted. The EXAFS investigations have shown that the majority of Fe ions are incorporated in tetrahedrally coordinated Ga sites in the lattice. The Mössbauer effect measurements have shown the existence of three kinds of Fe centers, two of them tetrahedrally coordinated in the LiGaO_2_ phase, the most intense one ascribed to isolated Fe centers within the nanocrystals, and the less intense one to Fe close to the surface. The trace amount of Fe found in octahedral coordination is ascribed to Fe on octahedral Ga sites in the impurity LiGa_5_O_8_ phase, detected at 0.8% in X-ray studies.

In photo- and thermo-stimulated luminescence, the dominant emission stems from isolated Fe ions with ZPL at 709 nm. This center is efficiently excited with the above band-gap irradiation, in contrast to the Fe-defect complex with ZPL at 695 nm, for which intra-center excitation is much more efficient. A closer inspection of the PL spectra under high energy excitation reveals the presence of another zero phonon line at 657.6 nm, with an intensity equal to that of the 695 nm ZPL. This finding perfectly agrees with the results of EPR investigations, where three different Fe centers with orthorhombic symmetry were detected. The intensities cannot be directly compared. While the EPR signal intensity is proportional to concentration, the signal intensity depends on excitation efficiency in PL.

Our study also shows the importance of annealing conditions for obtaining a pure LiGaO_2_ phase. Although only a very small amount of impurity LiGa_5_O_8_ was detected after annealing at 1150 °C, there is a chance that even more precisely chosen temperature and time of annealing would lead to a more one-phase composition of the material. Certainly, this could be the subject of further investigations.

## Figures and Tables

**Table 1:** Comparison of Raman peaks in the main LiGaO_2_:Fe^3+^ and the impurity LiGa_5_O_8_:Fe^3+^ phases with previous Raman studies of undoped LiGaO_2_ and LiGa_5_O_8_ powder samples.

LiGaO_2_:Fe^3+^ (cm^−1^)	LiGa_5_O_8_:Fe^3+^ (cm^−1^)	LiGaO_2_ [13]	LiGaO_2_ [14]	LiGa_5_O_8_ [14]
129	128.4	128.7		
139.2	135.1			
202		204.2		
219				
249.5		252.1	252	
	271.3			270
287.2		289.0	289	
318.6	344			344
397	371			372
	425			424
435		444.3	443	
495.3	502.8	502.1	501	512
556	578			579
	605.4			605
640		643.9	643	
649	654	653.8	655	655
714				
760	760		763	763

## Data Availability

All data regarding this work are included in the main article and the Supplementary Information.

## Supplementary Materials

The following supporting information can be downloaded at: https://www.mdpi.com/article/10.3390/molecules30112331/s1, Text S1: Antimicrobial properties; Figure S1: a: FE SEM micrograph of the as-grown Fe^3+^ doped LiGaO_2_ sample; b. EDS Elemental map of LiGaO_2_:Fe^3+^ sample annealed at 1450 °C; Figure S2: Vacuum UV Luminescence and Afterglow; Figure S3: Pressure dependence of the 695 nm emission line intensity; Figure S4: Low temperature luminescence spectra of the sample annealed at 1150 °C, containing mainly LiGaO_2_ phase, and the sample annealed at 1450 °C, with a large amount of the additional LiGa_5_O_8_ phase.

## Abbreviations

The following abbreviations are used in this manuscript:

CTB	Charge transfer band
CL	Cathodoluminescence
EPR	Electron paramagnetic resonance
EXAFS	Extended X-ray absorption fine structure
EDS	Energy Dispersive Spectroscopy
FE-SEM	Field Emission Scanning Electron Microscopy
NIR	Near-infrared
PL	Photoluminescence
VUV	Vacuum UV
XAS	X-ray absorption spectroscopy
XRD	X-ray diffraction
ZPL	Zero phonon line
