# Supplementary material for: Multisite Fe3+ Luminescent Centers in the LiGaO2:Fe Nanocrystalline Phosphor
Source: Molecules. 2025 May 27;30(11):2331. doi: 10.3390/molecules30112331 (PMC12155774; doi:10.3390/molecules30112331)
Supplement: Supplementary file 1 [file molecules-30-02331-s001.zip › molecules-3567916-supplementary.pdf]

Supplementary Information

Multisite Fe^{3+} luminescent centers in the $LiGaO_2$:Fe nanocrystalline phosphor

**Ajeesh K. Somakumar[1], Ivo Romet[2], Agnieszka Grabias[3], Marcin Kruk[4], Shusaku Hayama[5], Damian Wlodarczyk[1], Justyna Barzowska[6], Yadhu K. Edathumkandy[1], Eduard Feldbach[2], Puxian Xiong[7], Yaroslav Zhydachevskyy[1], Monika Trzaskowska[4], Hanka Przybylińska[1], and **Andrzej Suchocki [1]*

[1]Institute of Physics, Polish Academy of Sciences, Aleja Lotników 32/46, 02-668 Warsaw, Poland

[2]Institute of Physics, University of Tartu, W. Ostwald Str. 1, 50411 Tartu, Estonia

[3]Łukasiewicz Research Network – Institute of Microelectronics and Photonics, Aleja Lotników 32/46, 02-668 Warsaw, Poland

[4] Institute of Human Nutrition Sciences, Warsaw University of Life Sciences Nowoursynowska 159c, 02-776 Warsaw, Poland.

[5]Diamond Light Source, Harwell Science & Innovation Campus, Didcot OX11 DE, U.K.

[6]Institute of Experimental Physics, Faculty of Mathematics, Physics and Informatics, University of Gdansk, Wita Stwosza 57, 80-952 Gdańsk, Poland

[7]Department of Electrical and Electronic Engineering, The University of Hong Kong, Hong Kong 999077, China

*Ajeesh K. Somakumar, e-mail: skumar@ifpan.edu.pl

**Andrzej Suchocki, e-mail: suchy@ifpan.edu.pl;

Text S1: Antimicrobial properties

The near-infrared emitting $LiGaO_2$: Fe^{3+} phosphor has been recently shown to exhibit excellent persistent luminescence properties which, together with the reported cytotoxicity tests on biological tissues, indicate its potential for bio-imaging applications [1]. This inspired us to check, whether the material shows also antibacterial properties. We found that in the as grown form it does not exhibit any significant antibacterial action against the following gram-positive and gram-negative bacteria investigated: ***Salmonella enterica* ATCC 29631, *Salmonella enterica* ATCC 14028, *Escherichia coli* ATCC 15922, *Escherichia coli* ATCC 11775, *Listeria monocytogenes* ATCC 15131, *Listeria monocytogenes* ATCC 7644**. This may change if a suitable capping is introduced. The experimental method used for the antimicrobial studies is described below.

The agar-based well diffusion method was used to analyse the antimicrobial properties of nanoparticles. Initially, *Salmonella enterica* ATCC 29631, *Salmonella enterica* ATCC 14028, *Escherichia coli* ATCC 15922, *Escherichia coli* ATCC 11775, *Listeria monocytogenes* ATCC 15131, *Listeria monocytogenes* ATCC 7644 were activated at 80°C. Bacterial strains were activated on a Mueller Hinton Agar plate (Oxoid, UK) and incubated aerobically for 24 h at 37°C. Then one colony was transferred to 10 mL of Mueller-Hinton broth (Oxoid, UK) and incubated overnight at 37 °C. Then the cultures were serially diluted in phosphate-buffered saline (Sigma-Aldrich, Poznań, Poland) to obtain bacteria concentration between 4-5 log CFU/mL. On each 90 mm diameter Petri dish, 25 mL of the Muller Hinton Agar was poured and solidified before culturing. Bacterial supersites were cultured in the volume of 100 μL and spread on the surface of agar plates. Wells were cut in the agar using a 6 mm diameter knife. A solution of nanoparticles in water was poured into each well in a volume of 100 μL. Two concentrations of nanoparticles were tested: 1 g/100 mL (1%) and 0.1 g/100 mL (0.1%). The plates were incubated for 24 hours at 37°C under aerobic conditions. The experiment was carried out in three biologically independent repetitions.

[1] Z. Zhou, X. Yi, P. Xiong, X. Xu, Z. Ma, M. Peng, Cr^{3+}-Free near-infrared persistent luminescence material $LiGaO_2$:Fe^{3+}: Optical properties, afterglow mechanism and potential bioimaging, J Mater Chem C Mater 8 (2020) 14100–14108. https://doi.org/10.1039/d0tc03212c.

S1: FE-SEM and EDS elemental mapping

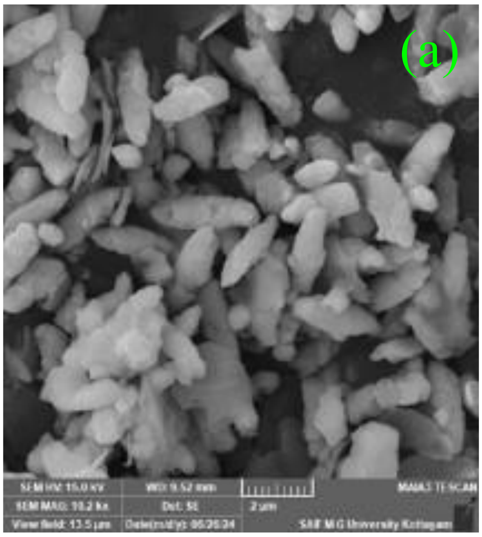

Figure S1(a). FE SEM micrograph of the as-grown Fe^{3+} doped $LiGaO_2$ sample.

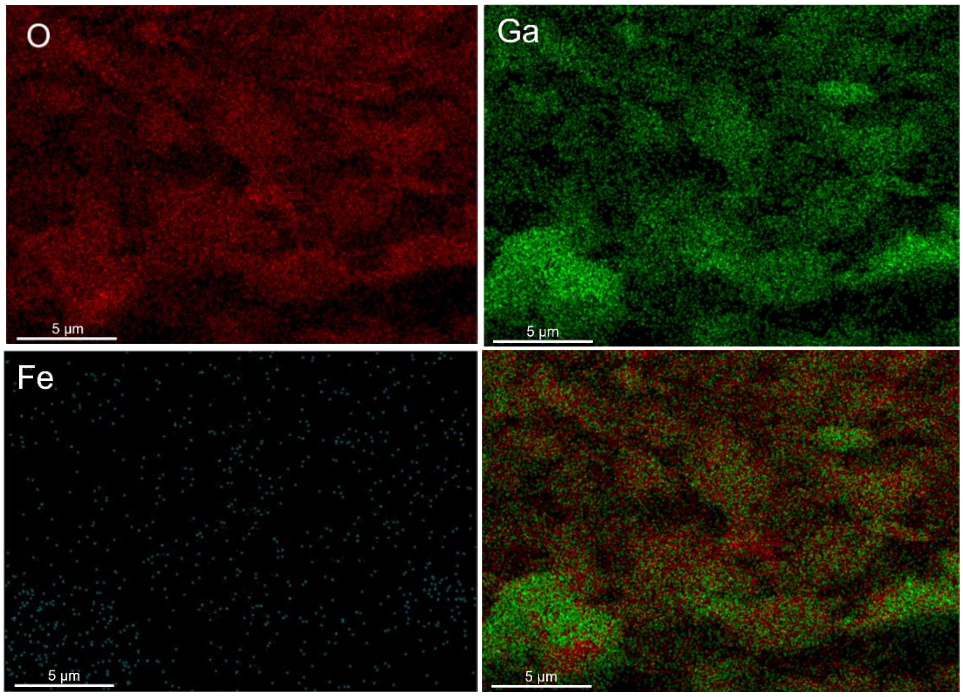

Figure S1(b). EDS Elemental map of $LiGaO_2$:Fe^{3+} sample annealed at 1450 °C: O – oxygen, Ga – gallium, Fe – iron.

S2: Vacuum UV Luminescence and Afterglow

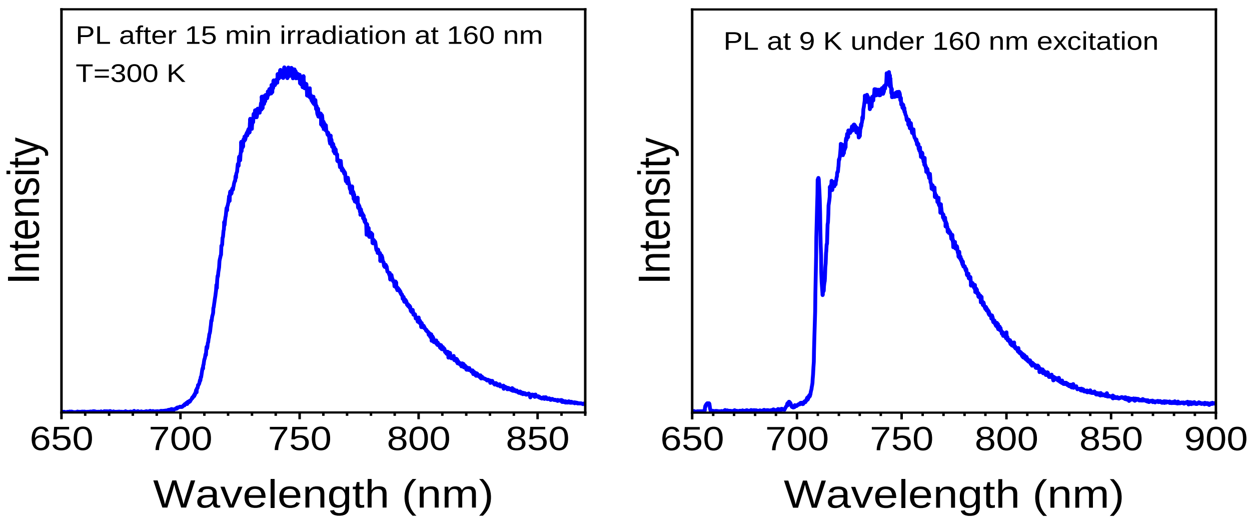

Figure S2. Afterglow after exposure to 15 min irradiation at 160 nm detected at room temperature (left) and luminescence under the same excitation at 9 K (right).

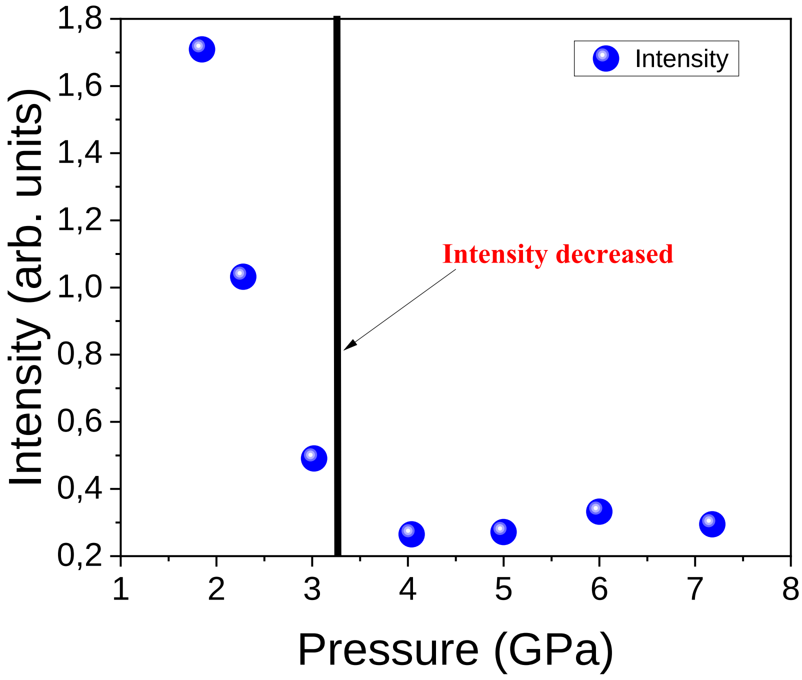

Figure S3. Pressure dependence of the 695 nm emission line intensity.

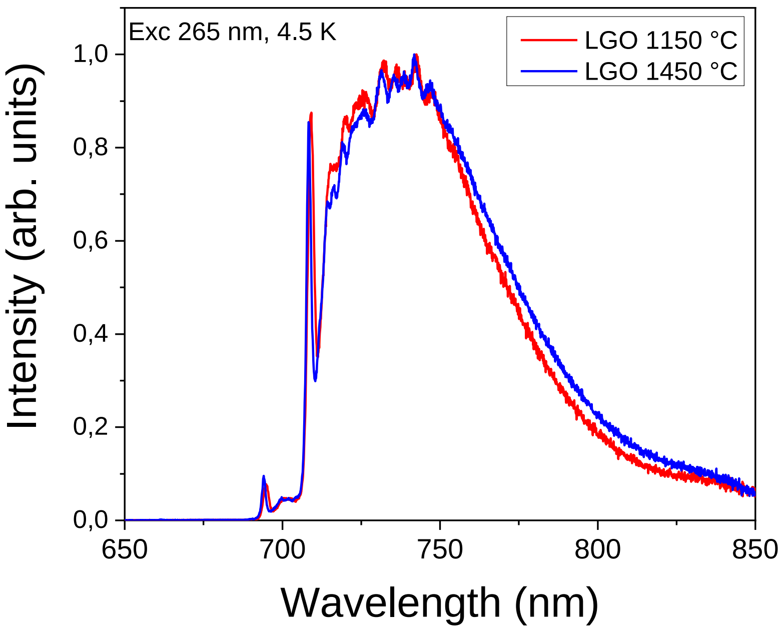

Figure S4. Low temperature luminescence spectra of the sample annealed at 1150 ^{0}C, containing mainly $LiGaO_2$ phase, and the sample annealed at 1450 ^{0}C, with a large amount of the additional $LiGa_5O_8$ phase.
